# A Powassan virus domain III nanoparticle immunogen elicits neutralizing and protective antibodies in mice

**DOI:** 10.1371/journal.ppat.1010573

**Published:** 2022-06-09

**Authors:** Ryan J. Malonis, George I. Georgiev, Denise Haslwanter, Laura A. VanBlargan, Georgia Fallon, Olivia Vergnolle, Sean M. Cahill, Richard Harris, David Cowburn, Kartik Chandran, Michael S. Diamond, Jonathan R. Lai

**Affiliations:** 1 Department of Biochemistry, Albert Einstein College of Medicine, Bronx, New York, United States of America; 2 Department of Microbiology and Immunology, Albert Einstein College of Medicine, Bronx, New York, United States of America; 3 Department of Medicine, Washington University in St. Louis, School of Medicine, St. Louis, Missouri, United States of America; 4 Department of Molecular Microbiology, Washington University in St. Louis, School of Medicine, St. Louis, Missouri, United States of America; 5 Department of Pathology & Immunology, Washington University in St. Louis, School of Medicine, St. Louis, Missouri, United States of America; Colorado State University, UNITED STATES

## Abstract

Powassan virus (POWV) is an emerging tick borne flavivirus (TBFV) that causes severe neuroinvasive disease. Currently, there are no approved treatments or vaccines to combat POWV infection. Here, we generated and characterized a nanoparticle immunogen displaying domain III (EDIII) of the POWV E glycoprotein. Immunization with POWV EDIII presented on nanoparticles resulted in significantly higher serum neutralizing titers against POWV than immunization with monomeric POWV EDIII. Furthermore, passive transfer of EDIII-reactive sera protected against POWV challenge *in vivo*. We isolated and characterized a panel of EDIII-specific monoclonal antibodies (mAbs) and identified several that potently inhibit POWV infection and engage distinct epitopes within the lateral ridge and C-C′ loop of the EDIII. By creating a subunit-based nanoparticle immunogen with vaccine potential that elicits antibodies with protective activity against POWV infection, our findings enhance our understanding of the molecular determinants of antibody-mediated neutralization of TBFVs.

## Author summary

Powassan virus (POWV) is an emerging tick-borne flavivirus that circulates in North America. Infection can cause severe neuro-invasive disease, and currently there is no treatment or vaccine against POWV. We generated a novel POWV immunogen that displays the EDIII of the envelope glycoprotein on the surface of a self-assembling protein nanoparticle. We found that immunization of the EDIII-displaying nanoparticle in mice elicited potently neutralizing and protective antibodies. We further characterized EDIII-specific neutralizing mAbs and mapped their epitopes to specific regions within the POWV EDIII. Our study sheds light on determinants of antibody-mediated neutralization and protection against POWV and other TBFVs. It also informs EDIII-based vaccine and antiviral efforts to combat POWV infection.

## Introduction

Powassan virus (POWV) is an emerging tick-borne flavivirus (TBFV) that causes neurological disease in humans [1–4]. The first human infection was documented in Ontario, Canada in 1958, and the virus currently circulates in areas of Canada, the United States, and Russia. While neuroinvasive disease caused by POWV infection remains rare, there has been a steady rise in incidence in the United States in recent years, especially during 2016–2019 [3,5]. Neurological manifestations include encephalitis, meningitis, and meningoencephalitis [6]. Roughly half of those with neuroinvasive disease experience long-term neurological sequelae, and 10% of cases are fatal [7–9]. There are currently no approved vaccines or antiviral treatments for POWV infection.

POWV, like other TBFVs, is an enveloped, positive-sense RNA virus. There are two POWV lineages (lineage I, and lineage II or deer-tick virus) that share 96% amino acid sequence identity in the envelope (E) glycoprotein and are identical in terms of serology and clinical presentation [10,11]. POWV lineage I (POWV-I) and lineage II (POWV-II) are transmitted by distinct species of ticks, *Ixodes cookei* and *Ixodes scapularis*, respectively [12–14]. Incidence of tick-borne infections is thought to be increasing due to expansion of residential areas into forested regions [15] and global climate change, which results in a longer tick season [16]. Indeed, the geographical distribution of *Ixodes scapularis* in the United States has expanded in recent decades [17,18].

The flavivirus envelope (E) glycoprotein exists as a homodimer and contains three domains I, II, and III (EDI, EDII, and EDIII) [19–21]. The EDI includes the N-terminus of the E protein and forms an eight-stranded β-barrel structure that acts as a molecular hinge [22]. The EDII contains a dimerization domain as well as the fusion loop peptide that facilitates pH-dependent membrane fusion in the late endosome [23]. The EDIII is an Ig-like domain that facilitates interaction with host cells, undergoes large motions required during viral membrane fusion, and is a target of neutralizing antibodies for many flaviviruses [24–28].

Recent work toward POWV vaccine development, which includes the investigation of mRNA [29], DNA [30] and VLP [31] platforms, has focused on the presentation of POWV prM and E proteins as the antigen. While these platforms have demonstrated the induction of neutralizing and protective antibody responses in mice, the full prM and E proteins contain many antigenic sites, only a subset of which are targets of functional antibodies that limit POWV infection. Furthermore, it is known for other flaviviruses that certain conserved epitopes in EDII and prM elicit broadly reactive but poorly neutralizing antibodies, that can contribute to antibody-dependent enhancement (ADE) [32–34]. While the relevance of ADE in POWV is unknown, a vaccine containing only critical, functional epitopes sufficient to stimulate a protective antibody response would have advantages. While EDIII-based subunit vaccines have been explored for prevention of flaviviruses such as Dengue [35–37] and Zika virus [38–40], the immunogenicity of the POWV EDIII has not been determined.

Although recombinant subunit vaccines have shown promise, they can be hampered by the induction of weak or modest immune responses [41]. One emerging strategy is the presentation of viral antigens on self-assembling protein nanoparticles [42]. The ordered, multivalent display of viral structural proteins can effectively mimic a viral particle, resulting in a more robust immune response. Furthermore, multivalent display of antigens allows for greater efficiency of cross-linking B cell receptors, which is a critical step for B cell activation and antibody maturation [43]. This strategy has been successfully implemented for HIV [44], influenza [45], SARS-CoV2 [46], and other pathogens [47,48].

Recent work characterized several neutralizing POWV EDIII mAbs generated from mice immunized with an mRNA-based vaccine encoding POWV prM/E and identified epitopes on the EDII and EDIII as key targets of protective neutralizing POWV mAbs [26,29,49]. In particular, structural studies of the cross-neutralizing murine mAb POWV-80 revealed a complex lateral ridge/C-C’ loop epitope that included the N-terminus, C-C’, and FG loops. Still, few POWV mAbs that target the EDIII have been identified, and the critical epitope(s) within the EDIII targeted by neutralizing mAbs requires further elucidation.

Here, we characterize the POWV EDIII-specific antibody response in mice following immunization with a unique POWV immunogen that displays POWV EDIII on the surface of a self-assembling protein nanoparticle. Immunization with the POWV EDIII nanoparticle induced significantly higher neutralizing titers against POWV than immunization with the recombinant POWV EDIII alone in vaccinated mice, and the neutralizing EDIII-reactive sera protected against POWV challenge *in vivo*. We further defined the antibody response by isolating a panel of 12 mAbs by single B cell cloning that bind the POWV EDIII. We identified four mAbs that neutralize POWV and mapped their epitopes with a combination of competition, mutagenesis, and nuclear magnetic resonance (NMR) studies. Together, our work further defines the role of EDIII-specific antibodies in combatting POWV infection.

## Results

### Generation and characterization of recombinant POWV EDIII

**Fig 1:**
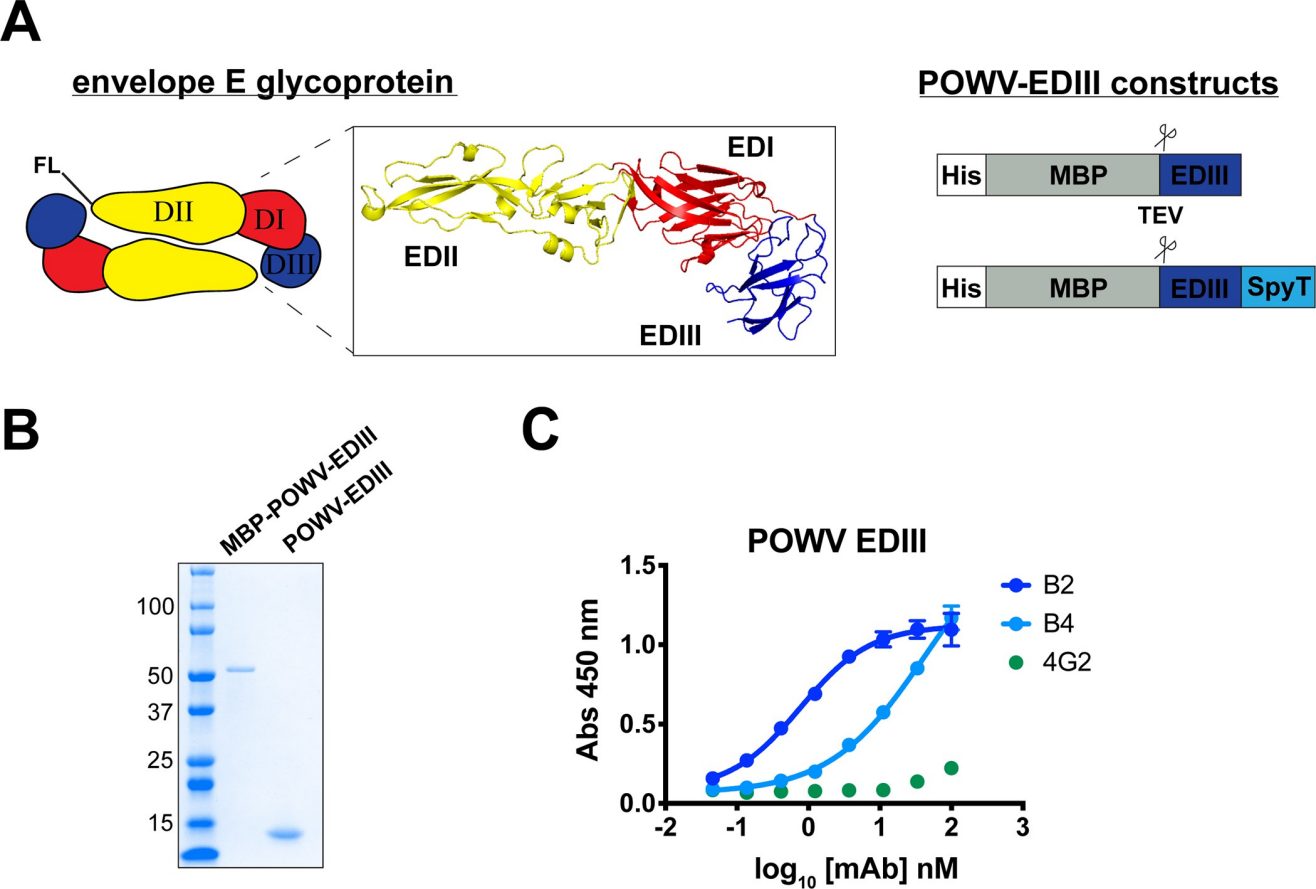
Design, expression, and purification of recombinant POWV EDIII. (**A**) Schematic of POWV E protein homodimer and POWV EDIII wild-type and Spy-tagged (SpyT) expression constructs. FL refers to fusion loop peptide contained in EDII. Inset shows structure of TBEV E glycoprotein (PDB: 1SVB). Expression constructs contain the POWV EDIII sequence as C-terminal fusion to His-tagged MBP and include a TEV protease cleavage site as indicated. (**B**) Coomassie stained SDS-PAGE gel of MBP-POWV-EDIII fusion protein and cleaved wild-type POWV EDIII. (**C**) ELISA reactivity of mAbs B2 and B4 toward POWV EDIII. MAb 4G2 is a negative control. ELISAs were performed twice independently in triplicate wells (mean ± SD).

To investigate the antibody response to POWV EDIII, we first designed constructs for soluble expression of the recombinant POWV EDIII in *E*. *coli*. In addition to the wild-type POWV EDIII, we designed POWV EDIII SpyT, which contains a C-terminal SpyTag [50] for subsequent covalent conjugation to a SpyCatcher-containing nanoparticle (**Fig 1A**). We expressed the POWV EDIII as a C-terminal fusion to maltose binding protein (MBP), which permitted soluble expression of the MBP-POWV-EDIII fusion protein in high yield (~50–70 mg/L). Notably, this strategy, which has been successful for other flavivirus EDIIIs, avoids insoluble expression from inclusion bodies that requires biochemical refolding [51]. Proteolytic cleavage of the MBP with tobacco etch virus (TEV) protease followed by anion exchange chromatography resulted in purified POWV EDIII, with yields of 5–8 mg/L (**Fig 1B**). To assess the antigenicity of POWV EDIII, we tested the reactivity of murine mAbs B2 and B4, which cross-react with tick-borne encephalitis virus (TBEV) [52,53] by enzyme-linked immunosorbent assay (ELISA). Both mAbs reacted with POWV EDIII, confirming that the recombinant protein adopts an antigenically relevant conformation **(Fig 1C**). Thus, this expression strategy affords efficient production of recombinant POWV EDIII for subsequent immunogenicity studies.

### Generation and characterization of a POWV EDIII nanoparticle immunogen

**Fig 2:**
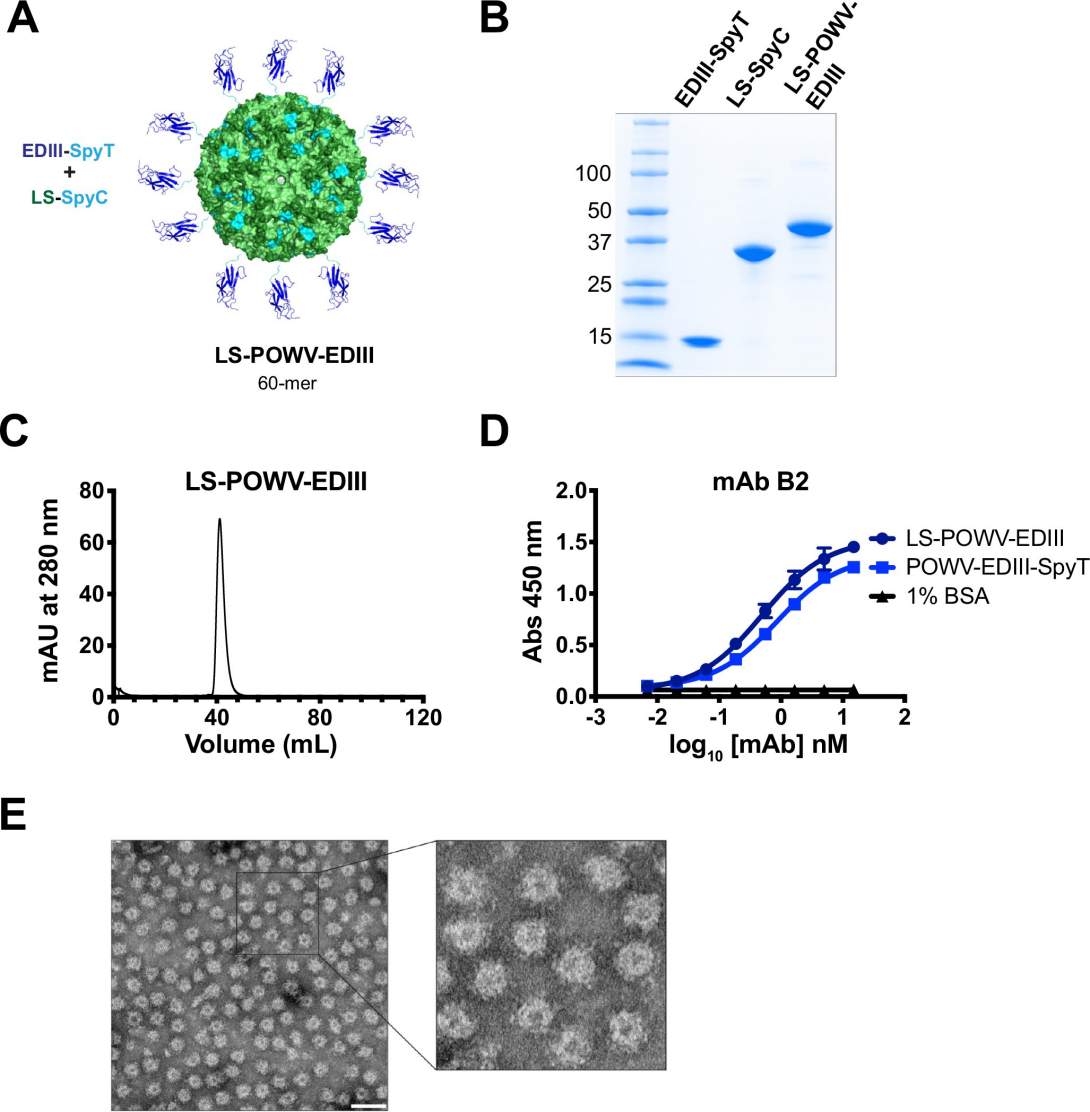
Production and characterization of LS-POWV-EDIII nanoparticle immunogen. (**A**) Schematic representation of LS-POWV-EDIII nanoparticle. Structures of LS (PDB: 1HQK; green) and POWV EDIII (blue) are covalently linked via SpyCatcher/SpyTag (cyan). (**B**) Coomassie stained SDS-PAGE gel of POWV EDIII nanoparticle conjugation using SpyCatcher/SpyTag system. Purified POWV EDIII-SpyT (12.6 kDa), LS-SpyC (32 kDa), and conjugated LS-POWV-EDIII (45 kDa) are shown. (**C**) Size exclusion chromatogram of LS-POWV-EDIII. Sample was run on Sephacryl S-400 HR AKTA column. A retention time of 45 mL was observed (MW 1x10^6^-1x10^7^ Da). (**D**) ELISA reactivity of mAbs B2 toward POWV EDIII-SpyT and LS-POWV-EDIII. ELISAs were performed twice independently in triplicate wells (mean ± SD). (**E**) Negative-stain transmission EM images of LS-POWV-EDIII. Scale bar is 50 nm.

We hypothesized that presentation of POWV EDIII on a nanoparticle platform would boost immunity. We used the SpyCatcher/SpyTag system [50,54] to covalently couple POWV-EDIII-SpyT to *Aquifex aeolicus* lumazine synthase (LS) [55,56] that contained a C-terminal SpyCatcher sequence (LS-SpyC) (**Fig 2A**). LS is a spherical, multimeric enzyme involved in riboflavin synthesis that has been utilized as a nanoparticle platform for presentation viral antigens [44,55]. Purified LS-SpyC was incubated with POWV-EDIII-SpyT, and the purified LS-POWV-EDIII conjugate was assessed by SDS-PAGE. A polypeptide of ~45 kDa was observed under denaturing conditions, consistent with a single product containing POWV-EDIII-SpyT (12.6 kDa) and LS-SpyC (32 kDa) (**Fig 2B**). Each subunit of LS, a 60-mer, is thus conjugated to the EDIII, resulting in 60 copies of the POWV EDIII per particle. The expected molecular weight of LS-POWV-EDIII is ~2.6 x 10^6^ Da. Purification of LS-POWV-EDIII by size-exclusion chromatography demonstrated a single peak with a retention time consistent with a molecular weight of approximately 1x10^6^-1x10^7^ Da (**Fig 2C**).

To determine whether presentation on the nanoparticle altered the antigenicity of POWV EDIII, reactivity of the POWV-EDIII-SpyT monomer and the LS-POWV-EDIII nanoparticle to mAb B2 was tested by ELISA (**Fig 2D**). MAb B2 reacted with both monomeric and multivalently-displayed POWV EDIII, suggesting they shared similar conformations. We directly visualized LS-POWV-EDIII particles by negative-stain electron microscopy (nsEM) and observed spherical assemblies approximately ~20 nm in diameter (**Fig 2E**). The nanoparticles appeared uniform and monodispersed (non-aggregated). Thus, recombinant POWV EDIII can be readily conjugated and presented onto LS nanoparticles.

### Immunization of mice with LS-POWV-EDIII nanoparticles induces neutralizing antibodies

**Fig 3:**
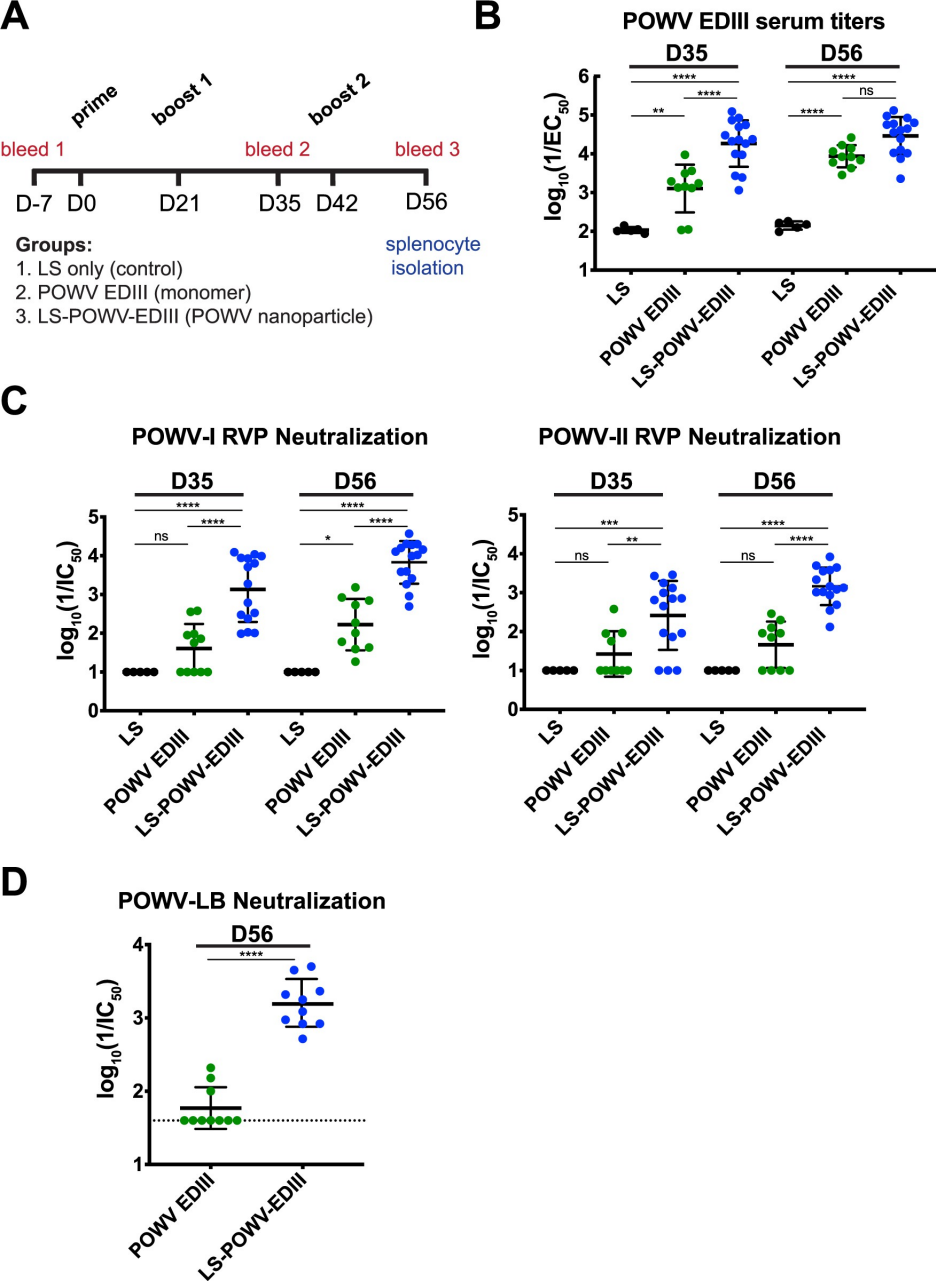
Binding and neutralization of antibodies induced by POWV EDIII immunogens. (**A**) Immunization schedule of mice with POWV EDIII immunogens. Mice were immunized on Day 0 (prime), Day 21 (1^st^ boost), and Day 42 (2^nd^ boost) with either LS (15 μg), POWV EDIII monomer (5 μg), or LS-POWV-EDIII (15 μg). Sera was collected 2 weeks after each boost as indicated. Splenocytes were isolated on Day 56 for mAb isolation. (**B**) ELISA reactivity of sera from immunized mice toward POWV EDIII. Groups shown are LS alone (n = 5), POWV EDIII monomer (n = 10) and LS-POWV-EDIII (n = 15) at day 35 and day 56. Full curve ELISAs were performed twice independently in triplicate wells (mean ± SD) and EC_50_ values were determined using non-linear regression analysis. (**C**) Serum neutralization of POWV-I (left) and POWV-II (right) RVPs from immunized mice. Groups shown are LS alone (n = 5), POWV EDIII monomer (n = 10) and LS-POWV-EDIII (n = 15) at day 35 and day 56. Neutralization curves were performed twice independently in triplicate wells (mean ± SD), and IC_50_ values were determined using non-linear regression analysis. (**D**) Serum neutralization of authentic POWV-LB strain by FRNT. Groups shown are POWV EDIII monomer (n = 10) and LS-POWV-EDIII (n = 10) at day 56. Full neutralization curves were performed twice independently in duplicate wells (mean ± SD), and IC_50_ values were determined using non-linear regression analysis. For B and C, one-way ANOVA with Tukey’s multiple comparison test was done. For D, unpaired, two-tailed t-test was done. (*, P < 0.05; **, P < 0.01; ***, P < 0.001, ****, P < 0.0001).

To evaluate the immunogenicity of POWV EDIII, we immunized mice with either LS alone, POWV EDIII monomer, or LS-POWV-EDIII nanoparticle (**Fig 3A**). We followed an immunization schedule that included an initial immunization (day 0) followed by two boosts at days 21 and 42; sera were collected two weeks after each boost. Dosing was adjusted based on the molecular weight of the antigen to ensure an equivalent number of moles of antigen for direct comparison (5 μg for POWV EDIII monomer; 15 μg for LS-POWV-EDIII). The squalene-based adjuvant AddaVax, which is similar in formulation to the licensed adjuvant MF59, was used for all immunizations.

We tested sera isolated from immunized mice at days 35 (two weeks after the first boost) and 56 (after second boost) for antigen reactivity towards POWV EDIII by ELISA and compared their half maximal effective concentrations (EC_50_ values) (**Fig 3B**). As expected, immunization with the unconjugated LS alone control did not elicit POWV EDIII-specific antibodies. Immunization with POWV EDIII monomer induced POWV EDIII directed antibodies at day 35 (mean EC_50_ 1:1250), but binding titers improved following the second boost at day 56 (mean EC_50_ 1:8500). In contrast, sera from LS-POWV-EDIII mice demonstrated >10-fold higher binding titers (mean EC_50_ 1:18,400) following a single boost at day 35 than the monomer group after a weight-adjusted equivalent dose. LS-POWV-EDIII titers were augmented at day 56 relative to day 35 (mean EC_50_ 1:28,800), but not significantly different than those of the POWV EDIII monomer after two boosts. Together, these results demonstrate that both POWV-EDIII monomer and LS-POWV-EDIII can elicit a robust EDIII-specific antibody response in mice, but additional immunization of the POWV-EDIII monomer was needed to achieve equivalent binding titers to the LS-POWV-EDIII nanoparticle immunogen.

We next characterized the nature of the polyclonal antibody response elicited by POWV-EDIII monomer and LS-POWV-EDIII by ELISA (**S1A Fig**). Antibody subclass profiles (IgG1, IgG2a, and IgG2b) induced by POWV-EDIII monomer and LS-POWV-EDIII were equivalent. Additionally, POWV-EDIII and LS-POWV-EDIII antisera reacted equally to monomeric POWV EDIII and multivalent LS-POWV-EDIII (**S1B Fig**). We also tested the cross-reactivity of POWV-EDIII and LS-POWV-EDIII antisera to related flavivirus EDIIIs, including TBEV, Langat virus (LGTV), and DENV2 (**S1C Fig**). Strong cross-reactivity of POWV-EDIII and LS-POWV-EDIII antisera was observed against TBEV and LGTV EDIIIs, but not DENV2.

Next, we tested serum neutralization by employing an established flavivirus-based reporter viral particle system (RVP) for POWV lineages I and II [57] (**Fig 3C**). POWV-I (lineage I) and POWV-II (lineage II) RVPs were constructed based on the C-prM-E proteins of strains POWV-LB and DTVWi99, respectively, and share 96% amino acid identity (**S2 Fig**). MAb B2 and B4 reactivity to POWV-I RVPs was confirmed by ELISA (**S3 Fig**). At day 35, LS-POWV-EDIII antisera was able to neutralize both POWV-I and POWV-II RVPs, with mean IC_50_ values of 1:1300 and 1:250, whereas the POWV EDIII antisera did not significantly neutralize either POWV-I or POWV-II. At day 56, serum neutralizing titers increased after the second boost in LS-POWV-EDIII immunized mice (POWV-I RVP 1:6200; POWV-II RVP mean IC_50_ 1:1100). The day 56 POWV EDIII antisera also showed increased neutralization of POWV-I RVP (mean IC_50_ 1:200) but not for POWV-II RVP.

Finally, we tested day 56 sera from both LS-POWV-EDIII and monomeric POWV EDIII groups against the authentic POWV-LB (lineage I) by focus reduction neutralization test (FRNT) (**Fig 3D**). LS-POWV-EDIII antisera neutralized authentic POWV-LB (mean IC_50_ 1:980) whereas POWV EDIII antisera showed minimal neutralizing activity. Notably, the mean IC_50_ of LS-POWV EDIII antisera against POWV-LB was (~7-fold) less potent compared to POWV-I RVP, consistent with previous studies [29]. Thus, we demonstrate that the LS-POWV-EDIII nanoparticle elicits a more potent and broad neutralizing antibody response in mice compared to the monomeric protein alone.

### Antibodies elicited by LS-POWV-EDIII protect against POWV-I infection in vivo

**Fig 4:**
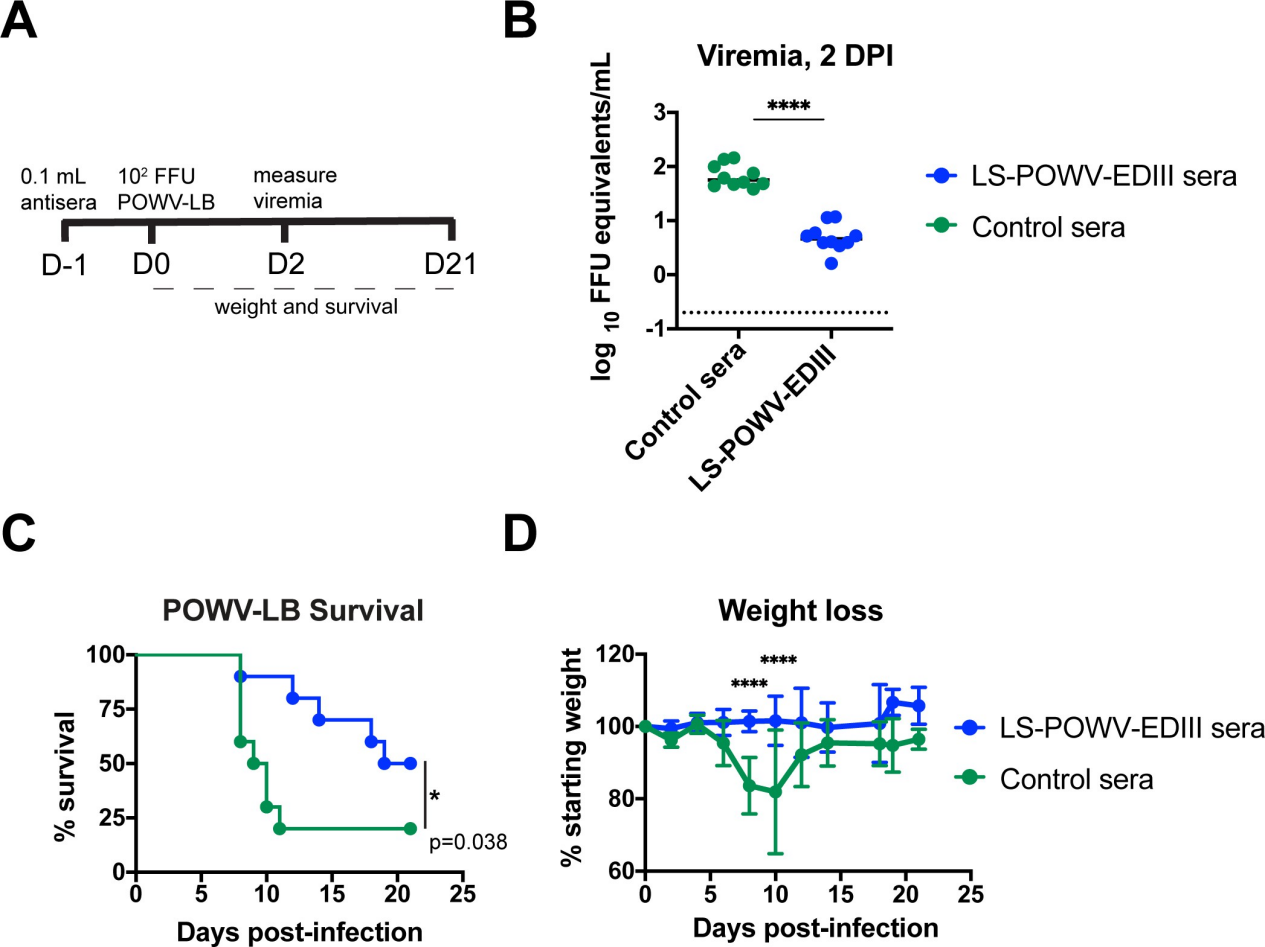
Serum from immunized mice protects against authentic POWV-LB challenge. **(A)** Timeline of passive serum transfer challenge. (**B**) Viremia, (**C**) survival, and (**D**) aggregate weight from passive serum transfer challenge against POWV-LB. 11-week-old C57BL/6J mice were administered 100 μL of pooled sera from naïve or LS-POWV-EDIII vaccinated mice and then challenged with 10^2^ FFU POWV-LB 24 hours later. Viremia was quantitated by qRT-PCR 2 days post-infection. Statistical analysis: (B) Two-tailed Mann Whitney test, (C) Log-rank Mantel-Cox test, and (D) Two-way ANOVA with Sidak’s multiple comparison test. (*, P < 0.05; ****, P < 0.0001).

We tested whether passive administration of LS-POWV-EDIII antisera could protect against lethal POWV-LB challenge (**Fig 4A)**. Mice treated with 0.1 mL of LS-POWV-EDIII antisera prior to POWV-LB challenge demonstrated a 14-fold decrease in viremia measured two days post-infection compared to mice treated with control sera (**Fig 4B**). Furthermore, mice treated with LS-POWV-EDIII antisera and challenged with POWV-LB showed enhanced survival times (50% survival vs. 20% in control-treated mice) and no weight loss compared to animals treated with control sera (**Fig 4C** and **4D**). Thus, antibodies elicited by LS-POWV-EDIII can neutralize authentic POWV and confer protection against lethal POWV challenge *in vivo*.

### Isolation of POWV EDIII-specific neutralizing mAbs

**Fig 5:**
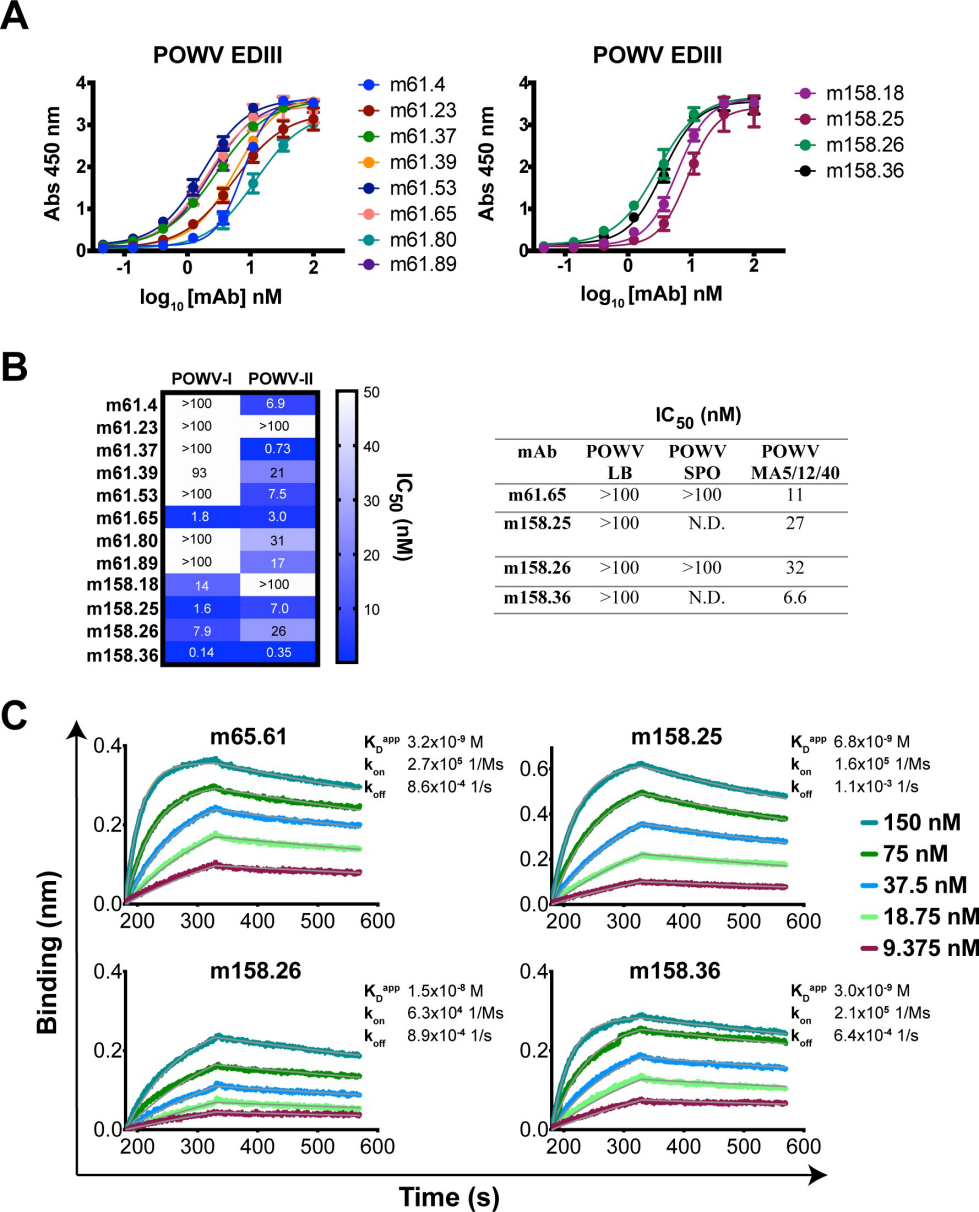
Binding and neutralization profiles of POWV EDIII mAbs. (**A**) ELISA reactivity of mAbs isolated from LS-POWV-EDIII immunized mice m61 (left) and m158 (right). Full curve ELISAs were performed twice independently in triplicate wells (mean ± SD). (**B**) Neutralization profiles of EDIII-reactive mAbs. A heatmap of IC_50_ values for 12 mAbs against POWV RVPs (left) and a table of IC_50_ values for the four most potent mAbs against authentic POWV strains (right) are shown. Neutralization curves were performed twice independently in triplicate wells (mean ± SD). (**C**) Binding of neutralizing mAbs to recombinant MBP-POWV by BLI. A representative dataset from two independent experiments is shown.

Since protective, EDIII-directed polyclonal antibodies were induced by LS-POWV-EDIII, we next isolated and characterized mAbs from immunized mice to further define the specific epitopes targeted by neutralizing antibodies. We generated mAbs by single B cell sorting from the splenocytes of two LS-POWV-EDIII immunized mice (“m61” and “m158”) to further interrogate the EDIII-specific neutralizing antibody response. We sorted individual CD19^+^ IgG^+^ POWV EDIII^+^ B cells and cloned the mouse variable domains as chimeric mAbs into a human IgG1 constant domain (**S4 Fig**). We identified 12 chimeric mAbs that strongly reacted with POWV EDIII by ELISA, with EC_50_ values ranging from 1.7 to 12 nM (**Fig 5A**). These mAbs had unique, unrelated sequences from diverse V-gene families (**S1 Table**). We then tested the panel of EDIII-reactive mAbs for neutralizing activity against POWV-I and POWV-II RVPs (**Fig 5B**). Full neutralization curves revealed a range of neutralizing potencies with IC_50_ values ranging from 0.15–580 nM and 0.35–33 nM, respectively for POWV-I and POWV-II. We identified four mAbs, m61.65, m158.25, m158.26, and m158.36, that potently neutralized both POWV-I and POWV-II RVPs, with IC_50_ values in the low- to sub-nanomolar range. These mAbs also neutralized the authentic POWV lineage II MA5/12/40 strain by FRNT, with IC_50_ values of 5.5–39 nM, but did not efficiently neutralize POWV-LB or POWV-SPO (**Fig 5B)**. Although somewhat unexpected, this result is consistent with previous findings that POWV lineage II MA5/12/40 strain, which contains a K10N mutation in the M protein that alters the display of E protein epitopes and is more sensitive to antibody neutralization [49].

To determine the binding kinetics of the four most potent POWV neutralizing mAbs against POWV-EDIII, we used biolayer interferometry (BLI). MAbs were captured on anti-human Fc sensors and then added to serially diluted MBP-POWV-EDIII. We observed that mAb binding to the MBP-POWV-EDIII fusion protein was more amenable to detection by BLI, likely due to its increased molecular weight relative to the EDIII alone. While sensorgrams could be fit with a 1:1 binding model, we cannot rule out avidity effects since the IgG is bivalent and refer to dissociation constants derived from on and off rates (*k*_*on*_ and *k*_*off*_) as “apparent” (*K*_*D*_^*app*^). The four neutralizing mAbs m61.65, m158.25, m158.26, and m158.36 all bound the MBP-POWV EDIII, with *K*_*D*_^*app*^ values ranging from 3.0–15 nM (**Fig 5C**). Thus, the most potently neutralizing mAbs all bound the EDIII subunit avidly with similar kinetic profiles.

### Epitope mapping of neutralizing POWV EDIII mAbs

**Fig 6:**
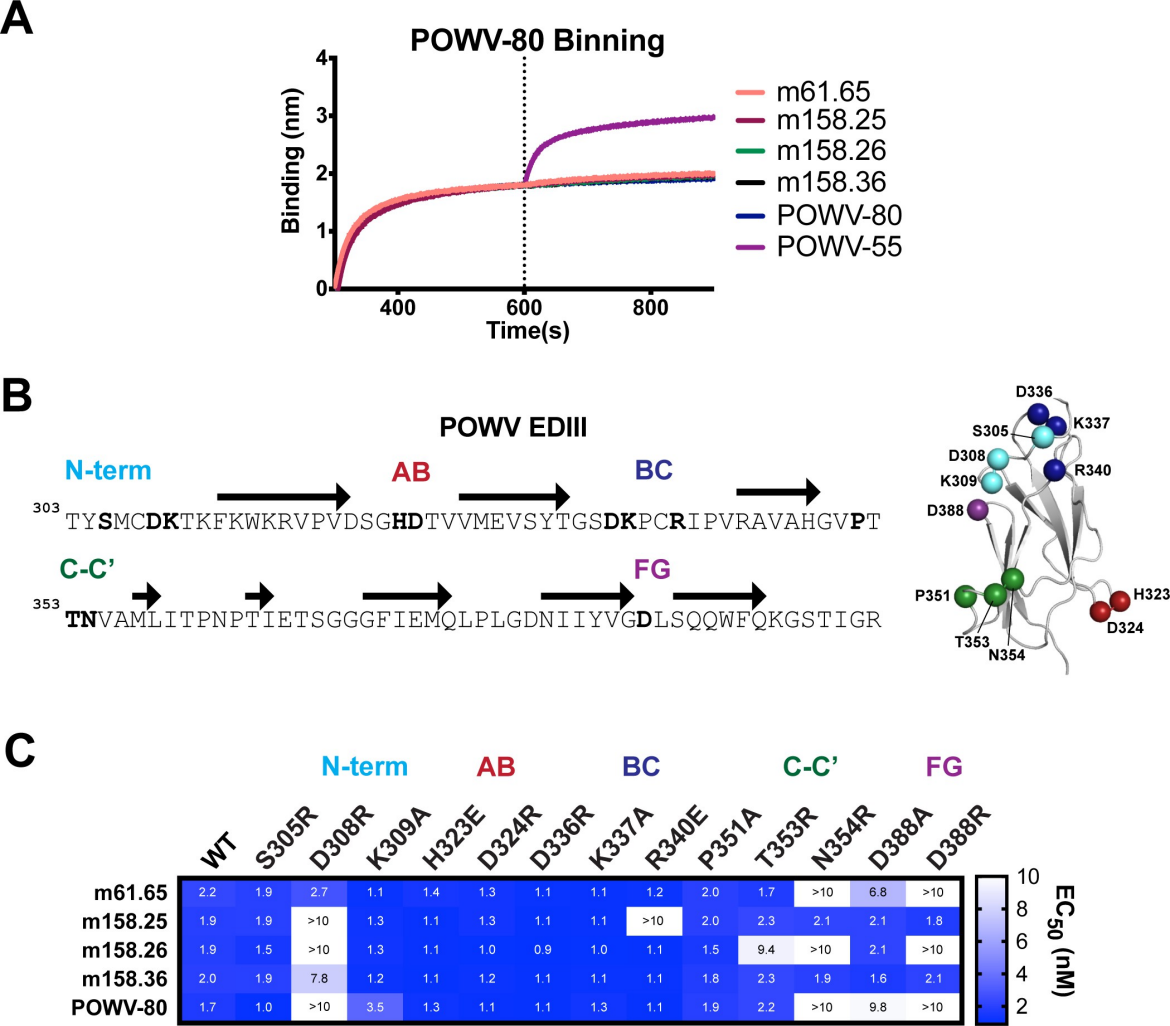
Epitope binning and mutagenesis mapping of neutralizing POWV EDIII mAbs. (**A**) Two-phase binding of neutralizing mAbs to POWV EDIII against mAb POWV-80 by BLI. MAb POWV-80 was bound to a sensor loaded with POWV EDIII followed by the sequential addition of the indicated second mAb. Representative data from two independent experiments is shown. (**B**) Sequence map of POWV EDIII for mutagenesis studies. Bolded residues in N-terminus, AB, BC, C-C’ and FG loops indicate positions where substitutions were introduced. Structure of POWV EDIII with mutations highlighted as Cα spheres is shown. (**C**) Heatmap of EC_50_ values of mAbs against MBP-POWV-EDIII mutants. EC_50_ values were calculated using non-linear regression analysis of ELISA binding curves performed twice independently in triplicate wells (mean ± SD).

Recent work identified two discrete epitopes that are engaged by neutralizing antibodies within the POWV EDIII: the C-C’ loop targeted by mAb POWV-80 and the A-strand targeted by mAb POWV-55 [49]. To determine whether the neutralizing mAbs we isolated share similar epitopes, two-phase epitope binning was performed by BLI (**Figs 6A** and **S5**). POWV-80 was loaded onto sensors coated with POWV-EDIII and then added to an equimolar mixture of POWV-80 and mAbs m61.65, m158.25, m158.26, and m158.36. All neutralizing mAbs tested showed minimal binding to the POWV EDIII pre-loaded with POWV-80, indicating competition and engagement of a similar epitope. In contrast, mAb POWV-55 bound the POWV-80/EDIII complex as expected because their two epitopes are spatially distinct.

We tested the cross-reactivity of neutralizing mAbs to TBEV, LGTV, and DENV2 EDIIIs by ELISA (**S6 Fig**). Whereas mAbs m61.65, m158.25, m158.26, and m158.36 reacted with TBEV, none of these mAbs recognized the distantly related DENV2. Notably, m61.65 and m158.26 reacted similarly with LGTV, although m158.25 and m158.36 did not. Thus, epitopes targeted by m61.65 and m158.26 are conserved among POWV, TBEV and LGTV, and m158.25 and m158.36 bind epitopes that show less cross-reactivity.

To identify the key residues that comprise the epitopes of POWV neutralizing mAbs, we generated a panel of single point mutants of the MBP-POWV-EDIII and tested mAb reactivity by ELISA. Substitutions were made at twelve positions within the AB, BC, C-C’, and FG loops as well as the N-terminus (**Fig 6B)**. Importantly, the introduction of mutations did not appear to globally alter protein conformation or stability, as evidenced by POWV-55 reactivity to all mutations tested (**S7 Fig**). MAbs m61.65, m158.25, m158.26, m158.36 and POWV-80 exhibited altered reactivity to the MBP-POWV-EDIII point mutants (**Figs 6C and S8**). Notably, N-terminal mutation D308R reduced reactivity to m158.25, m158.26, m158.36 and POWV-80. Mutations in the AB and BC loops generally did not impact binding, except for R340E in the BC loop, which caused a specific, complete loss of reactivity to m158.25. The C-C’ loop point mutation N354R as well as FG loop mutation D388R ablated binding to mAbs m61.65, m158.26, and POWV-80, but not mAbs m158.25 and m158.36. These data are consistent with the determined structural epitope of POWV-80, which shows engagement of the N-terminus, C-C’ loop, and FG loop. While the reactivity profiles of mAbs m61.65 and m158.26 appear similar to POWV-80, mAbs m158.25 and m158.36 were distinct, suggesting that different residues might be important in mediating POWV EDIII recognition for these mAbs. In particular, m158.25 seems to engage a region of the BC loop that is functionally important for the antibody-antigen interaction.

### POWV EDIII solution structure and m158.25 epitope mapping by NMR

**Fig 7:**
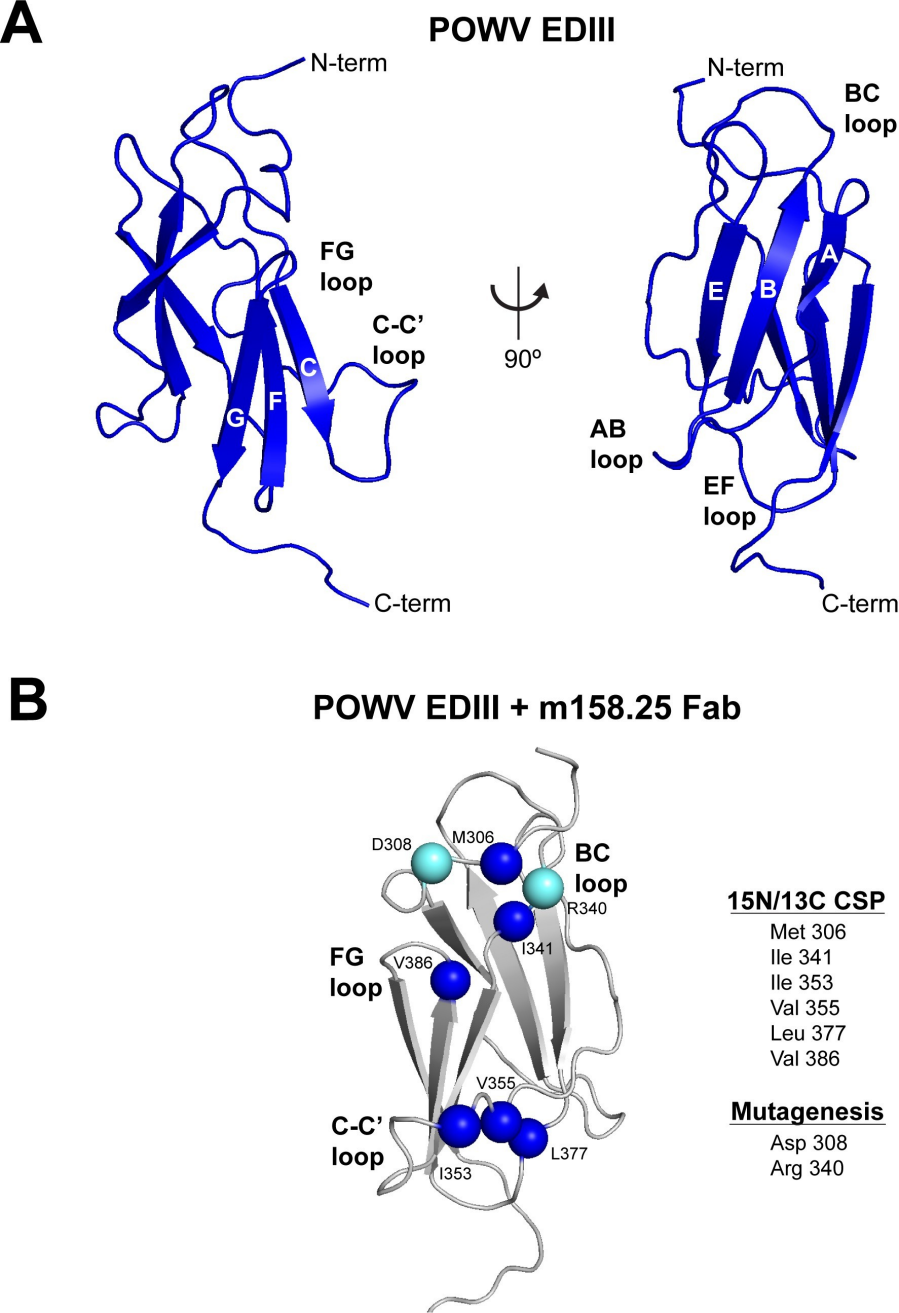
Solution structure of POWV EDIII and epitope mapping of m158.25 by NMR. (**A**) Ribbon display of lowest-energy structure of ^15^N/^13^C-labeled POWV EDIII determined by NMR. POWV EDIII contains six antiparallel β-strands in an Ig-like fold. β-strands and loop regions are indicated. (**B**) Epitope mapping of m158.25 by NMR. Methyl and amide signal changes induced by complexing ^15^N/^13^C-labeled POWV EDIII with m158.25 Fab were detected and positions identified were mapped to NMR structure as Cα spheres (blue). Residues that impacted m158.25 binding in mutagenesis scan are also highlighted (Cα spheres, cyan).

To gain additional insight into the antigenicity of POWV EDIII, we determined the solution structure by protein NMR spectroscopy using ^15^N/^13^C-labeled POWV EDIII (**Figs 7A and S9**). The three-dimensional solution structure of POWV EDIII contains six antiparallel β-strands forming a β-barrel-type structure, which resembles the immunoglobulin constant domain. The six β-strands are components of strands A through G and span the residues 313–317 (A), 326–332 (B), 343–347 (C), 370–375 (E), 381–386 (F), 389–394 (G), and a disulfide bridge exists between Cys307 and Cys339. The solved POWV EDIII solution structure was consistent with the X-ray structure recently solved in complex with POWV-80 antigen binding fragment (Fab) [49].

We next mapped the epitope of m158.25 in complex with the POWV EDIII by NMR (**Fig 7B**). We complexed the ^15^N/^13^C-labeled POWV EDIII with m158.25 Fab at a 1:1.1 stoichiometry and monitored both amide and methyl shifts resulting from complex formation. We tested the monovalent binding kinetics of the m158.25 Fab to the POWV EDIII by BLI (**S10 Fig**) and determined that the kinetic profile was similar to that of the full length mAb with MBP-POWV-EDIII (K_D_ 20 nM versus K_D_^app^ 6.8 nM). We identified both amide and methyl peak shifts at six positions that were present in the EDIII/Fab complex by NMR. These localized to the N-terminus as well as BC, C-C′, EF, and FG loops. This agrees with m158.25 sharing a competition group with POWV-80 and with our mutagenesis mapping that determined functional residues (D308 and R340), which are proximal to contacts identified by NMR (M306 and I341) in the N-terminus and BC loop. Additionally, NMR identified EF loop residue I377 as an interaction residue, which is proximal spatially to the FG loop contacts. Thus, we further defined the binding “footprint” of m158.25 by NMR and confirmed it engages a lateral ridge/C-C′ loop epitope that includes novel regions of the BC and EF loops.

## Discussion

Although immunogenicity of viral antigens for other flaviviruses has been characterized, less is known about the humoral immune response to POWV. Recent work has identified EDIII-specific antibodies that can neutralize and protect against POWV infection in mice, but the targeted induction of such antibodies has not been explored. To enhance our understanding of POWV antibodies targeting the EDIII, we designed a POWV EDIII-based nanoparticle immunogen and characterized the neutralizing antibody response in mice.

Immunogenicity of recombinant EDIIIs has been explored in other flaviviruses including TBEV [58], WNV [59], and DENV [36]. In most cases, only modest antibody titers were observed, likely due to the rapid clearance and poor immunogenicity of small peptide and protein antigens. To circumvent this limitation, we utilized LS, a well-studied self-assembling protein nanoparticle, for multivalent display of POWV EDIII. Nanoparticle immunogens have been shown in other contexts to augment immunogenicity of recombinant proteins by extending half-life, increasing lymphatic trafficking [60], and enhancing B cell receptor cross-linking to promote B cell activation [61]. Although immunization with LS-POWV-EDIII resulted in a more rapid onset of high antigen-binding titers than the monomer alone, binding titers following the second boost were equivalent among groups receiving nanoparticle and monomeric POWV EDIII. In contrast, serum neutralization was markedly different, with 43-fold higher mean neutralization titers for LS-POWV-EDIII antisera against POWV-I RVPs than monomeric POWV EDIII antisera. Furthermore, while LS-POWV-EDIII antisera against heterologous POWV-II RVPs were approximately five-fold less potent than POWV-I RVPs, sera from mice immunized with POWV EDIII monomer failed to significantly neutralize the POWV-II RVP. Thus, presentation of POWV EDIII on the nanoparticle elicited antibodies with increased neutralizing activity compared to those induced by monomeric EDIII, even though both sets of sera had equivalent binding titers. While the reason for this difference is unknown, one explanation is that presentation of the EDIII in nanoparticle format may restrict exposure to certain epitopes that are targeted by neutralizing antibodies. These results demonstrate that the LS-POWV-EDIII immunogen affords improved functional activity of EDIII-targeted antibodies in terms of neutralizing potency and breadth.

Our data also show differences in antibody neutralization against POWV-I and POWV-II RVPs, despite high sequence similarity, which may be important to consider in POWV vaccine design. This may be explained in part due to strain-specific amino acid sequence differences in the EDIII. We also observed greater neutralizing potency of LS-POWV-EDIII antisera against the POWV-I RVP than the authentic POWV1-LB, even though they shared identical prM and E glycoprotein amino acid sequences. This discrepancy, which is consistent with previous work, may be due to several factors, including differences in maturation state or conformational dynamics of the viral particles [49,62,63].

Passive transfer of sera from LS-POWW EDIII immunized mice decreased viremia and improved survival after lethal POWV challenge in mice. While only partial protection from mortality was observed, we nonetheless demonstrate that polyclonal EDIII-directed antibodies are sufficient to reduce mortality and viral burden upon POWV challenge. Several factors may be important in antibody-mediated protection against POWV. More potent neutralizing titers or higher doses of EDIII-directed sera may be required for complete protection. Antibodies targeting other critical epitopes, including those proximal to the fusion loop of EDII, also may be necessary to afford complete protection. Beyond neutralization, Fc-effector functions may enhance control and clearance of POWV infection, although this has not yet been explored.

Several recent studies have evaluated potential POWV vaccines. Both a lipid nanoparticle (LNP)-encapsulated modified mRNA vaccine and a synthetic DNA vaccine encoding the POWV prM and E genes elicited potently neutralizing antibodies in serum and conferred protection in lethal challenge models in mice [29,30]. Another study demonstrated that a POWV virus-like particle (VLP), which also included the POWV prM and E genes, elicited strong neutralizing antibody responses [31]. Our immunogen, based solely on the recombinant EDIII-subunit, elicited potently neutralizing and protective antibodies in mice. Subunit vaccines may be advantageous due to their safety, inexpensive production, and more targeted antibody generation based on key protective epitopes.

To further understand the determinants of antibody neutralization, we isolated a panel of mAbs from LS-POWV-EDIII mice. Recent work has identified and characterized POWV-directed mAbs, but still relatively few EDIII-directed POWV mAbs are known. We isolated 12 mAbs that reacted strongly to POWV EDIII and showed a range of neutralizing potential. LS-POWV-EDIII therefore stimulates the production of both weakly-neutralizing and potently-neutralizing EDIII-targeting antibodies. Four mAbs, m61.65, m158.25, m158.26, and m158.36, neutralized both POWV-I and POWV-II RVPs, with IC_50_ values in the sub- to low- nanomolar range.

To better define the sites within the EDIII targeted by neutralizing antibodies, we mapped the epitopes of the four most potent neutralizing mAbs, m61.65, m158.25, m158.26, and m158.36. The epitopes of these mAbs overlap with neutralizing mAb POWV-80 based on competition studies. POWV-80 binds regions within the EDIII that include the N-terminus as well as the C-C’ and FG loops [49]. Binding studies with a panel of POWV EDIII mutants suggest that m61.65 and m158.26 share functional contacts with POWV-80 in the C-C’ (N354) and FG (D388) loops, whereas residues important for EDIII-recognition by m158.25 and m158.36 are distinct. Such differences in antibody-antigen recognition may be due in part to the specific configuration of EDIII epitopes presented on the nanoparticle surface, which may be distinct from those displayed on subviral particles.

We further characterized the epitope of m158.25 by NMR, which identified six additional residues within the epitope that were perturbed by complexing with the Fab: M306 (N-term), I341 (BC loop), I353, V355 (C-C’ loop), L377 (EF loop), and V386 (FG loop). The NMR results are consistent with our findings that: (1) m158.25 competes with mAb POWV-80; and (2) EDIII mutants D308R and R340E, proximal to M306 and I341, ablate m158.25 binding. Although the three-dimensional structures of m158.25 in complex with the EDIII is not available, our data suggests that its epitope may partially overlap with or be adjacent to the POWV-80 epitope and includes distinct regions in the BC and EF loops. Thus, mAb m158.25 targets a related but distinct lateral ridge/C-C’ loop epitope. Differences in mAb engagement at the mAb-EDIII binding interface may impact neutralization potency and breadth. Furthermore, diverse EDIII-antibodies that target similar epitopes but require distinct functional contacts may limit viral escape. Our work thus further defines the POWV EDIII antibody response and improves our understanding of the determinants of antibody-mediated protection against POWV infection. Such information may inform vaccine design efforts for the targeted elicitation of protective antibodies against POWV.

## Materials & methods

### Ethics statement

All mouse immunization studies were carried out in accordance with the Guide for the Care and Use of Laboratory Animals of the National Institutes of Health after approval by the Institutional Animal Care and Use Committee at the Albert Einstein College of Medicine. Mouse challenge studies were approved and performed in accordance with Washington University Animal Studies Committee.

### Cell lines and viruses

Vero cells (CCL-81) were passaged in Dulbecco’s Modified Eagle Medium (DMEM) supplemented with 10% fetal bovine serum (FBS) and 100 U/mL penicillin-streptomycin. ExpiCHO-S cells (Gibco) were maintained in ExpiCHO expression media as per manufacturer’s instructions. Cells were maintained at 37°C in the presence of 5% CO_2_. POWV lineage I strain LB, [53] which was isolated originally from a human brain sample, was obtained from the World Reference Center for Emerging Viruses and Arboviruses (R. Tesh and S. Weaver, University of Texas Medical Branch). POWV lineage II strain SPO was isolated from an adult deer tick [64]. POWV lineage II strain MA51240 was isolated from ticks collected in Wisconsin [65]. Viral stocks were propagated in Vero cells and used at passages three and four.

### Reporter virus particle (RVP) generation

The structural proteins C, prM and E of POWV lineage I (Genbank: NC_003687.1) and POWV lineage II (Genbank: HM440558.1) were codon-optimized and synthesized in a pCAGGS vector for expression in human cells by Epoch Life Science, Inc. West Nile (WN) virus subgenomic replicon-expressing plasmid pcDNA6.2-WNIIrep-GFP/zeo was a generous gift from Dr. Ted Pierson (NIH, Bethesda, MD) [57]. For the production of POWV RVPs, 293FT cells (Thermo Fisher) were seeded into 15 cm plates using DMEM high glucose (Gibco) media supplemented with 10% heat inactivated FBS (Atlanta Biologicals), 1% P/S (Gibco) and 1% Q (Gibco). Cells were transfected with 12 μg of total DNA in a ratio 1:3 (POWV C-prM-E to WNV replicon) per plate. Media was exchanged after eight hours to low glucose DMEM (Gibco) supplemented with 5% FBS (heat-inactivated, Atlanta Biologicals) and 25 mM HEPES (Gibco). After three to four days at 37°C and 5% CO_2_, the RVP containing cell supernatant was harvested, and cell debris was removed by centrifugation for 15 min at 4,000 rpm at 4°C twice. The cleared viral supernatant was pelleted through a 2 mL 30% (v/v) D-sucrose in PBS (pH 7.4) cushion using a SW28 rotor (Beckman Coulter) in a Beckman Coulter Optima LE-80K ultracentrifuge for 4 h at 4°C at 28,000 rpm. The RVP pellet was resuspended overnight on ice in PBS (pH 7.4), aliquoted and stored at -80°C until further use.

### Expression and purification of proteins

POWV, TBEV, and LGTV (GenBank accession #AAL32154.1, ACU65515.1, and NP_740295.1, respectively) EDIII sequences (residues 303–402) were codon optimized for *E*. *coli* expression, synthesized (IDT) and cloned into pET His_6_ MBP (Addgene plasmid #29656). Wild-type, Spy-tagged, and mutant EDIII plasmids were transformed in BL21(DE3) cells (NEB), and protein expression was induced at OD_600_ of 0.6–0.8 with 0.4 mM IPTG for 18 h at 22°C. For NMR characterization, either [U-^15^N] or [U-^15^N,^13^C] samples were expressed in cells grown in M9 minimal media supplemented with ^15^NH_4_Cl and/or [U-13C] D-glucose. Cells were lysed by sonication in 50 mM Tris pH 8.0, 250 mM NaCl, and MBP-POWV fusion proteins were purified by Ni NTA chromatography and confirmed by SDS-PAGE. Proteolytic cleavage with TEV was done overnight (1:50 w/w), and cleavage products were dialyzed in low salt buffer (50 mM Tris pH 9.0, 20 mM NaCl) before anion exchange with MonoQ 10/100 GL.

For LS-SpyCatcher, the *Aquifex aeolicus* lumazine synthase [56] was expressed in pET-28a vector with an N-terminal His tag in BL21(DE3) cells. Protein expression was induced at OD_600_ of 0.6–0.8 with 0.4 mM IPTG for 18 h at 22°C. Cells were lysed by sonication in 50 mM Tris pH 8.0, 250 mM NaCl, and LS-SpyC was purified sequentially by Ni NTA and size exclusion chromatography with a Sephacryl S400 column. Coupling of the POWV-EDIII-SpyT to LS-SpyC was done for 16 h at 25°C at a 4:1 stoichiometry followed by purification with sephacryl S400 column and confirmation by SDS-PAGE.

### Negative stain electron microscopy

For nsEM imaging of LS-POWV-EDIII, 400 mesh, carbon only grids were plasma cleaned using a Tergeo-EM Plasma Cleaner (PIE Scientific, USA). LS-POWV EDIII nanoparticles were adsorbed onto grids for 10 min, after washing with dH_2_O. Samples were negatively stained with 1% uranyl acetate and viewed on a Tecnai 20 transmission electron microscope (ThermoFisher) at 120 kV.

### Mouse immunizations and sera collection

Female BALB/c mice (6–8 weeks-old) were obtained from Charles River Laboratories and housed in vented cages. Mice were immunized with 200 μL of either 15 μg of LS-POWV-EDIII nanoparticles or a 5 μg POWV-EDIII (mole-adjusted) by intraperitoneal injection using a 1 mL syringe (BD). Addavax adjuvant (Invivogen) was used in all immunizations as per the manufacturer’s instructions. The immunization schedule included an initial prime dose followed by two boosts spaced 21 days apart. Blood was collected by submandibular bleeds, one week prior to prime immunization and two weeks following each boost injection, in an SST tube (BD). Samples were incubated at room temperature for 1 h to coagulate, centrifuged for 2 min at 10,000 g, and sera was collected.

### Mouse mAb isolation

Isolation of murine mAbs was adapted as previously described [66–68]. Antigen-reactive B cells were isolated from mouse splenocytes by fluorescence activated cell sorting (FACS) with the following antibodies: anti-mouse CD3 (PE-Cy7), CD8 (PE-Cy7), F4/80 (PE-Cy7), Gr-1 (PE-Cy7) CD19 (Pacific Blue), IgG (FITC). POWV EDIII was biotinylated using EZ-Link NHS-PEG4-Biotin (Life Technologies) and detected with streptavidin conjugated phycoerythrin (Life Technologies). Cells were sorted into single PCR tubes, and cDNA was generated by RT-PCR. Nested PCR was performed with murine IgH- and IgK-specific primers and cloned into pMAZ vector [69] containing human IgG1 for sequencing and recombinant expression. Sequences were analyzed using IMGT/V-quest tool [70]. Chimeric mAbs were transiently transfected in ExpiCHO cells as per the manufacturer’s protocol (Gibco) and purified by protein A chromatography. The m158.25 Fab was expressed with a C-terminal His tag on the heavy chain and was transiently transfected in ExpiCHO cells and purified by Ni-NTA affinity chromatography.

### ELISA binding assay

To assess antibody reactivity by ELISA, POWV EDIII or LS-POWV-EDIII was coated on half-area 96-well high binding plates (Costar) at 200 ng/well. Wells were blocked with 1% BSA at 25°C for 2 h and washed 5 times with PBS-T (PBS pH 7.4, 0.05% Tween-20). Mouse sera or mAbs were diluted in PB-T (PBS pH 7.4, 0.2% BSA, 0.05% Tween) and incubated for 1 h at 37°C. Plates were washed and HRP-conjugated anti-mouse IgG (Sigma) for mouse sera or protein A (Life Technologies) for mAbs was added. For IgG subclass detection, HRP-conjugated anti-mouse IgG1, IgG2a, or IgG2b (Invitrogen) was used as secondary. After 1 h incubation at 37°C, plates were washed and developed using TMB (Thermo Fisher). Absorbance at 450 nm was measured on Synergy H4 Hybrid reader (BioTek).

### Binding kinetics by BLI

MAb binding kinetics to MBP-POWV-EDIII was measured by BLI using an OctetRed96 instrument (ForteBio). MAbs were immobilized on anti-human Fc capture sensors and global data fitting to a 1:1 binding model was used to estimate values for the *k*_*on*_ (association rate constant), *k*_*off*_ (dissociation rate constant), and *K*_*D*_^*app*^ (apparent equilibrium dissociation constant). Data were analyzed using ForteBio Data Analysis Software 9. For the monovalent binding experiment with POWV EDIII, His-tagged Fab was immobilized on anti-Penta-His sensors and analyzed as described above for full-length mAbs. For two-phase binning experiments, biotinylated POWV EDIII was first loaded onto a streptavidin-coated sensor, and then the first mAb bound to saturation. The sensor was then added to a well containing the first and test mAbs at equimolar concentrations (50 nM).

### RVP neutralization assay

Mouse sera and mAbs were serially diluted in DMEM high glucose medium (Gibco), supplemented with 2% FBS (heat-inactivated, Atlanta Biologicals), 1% Q (Gibco), 1% P/S (Gibco) and 25 mM HEPES (Gibco), and incubated at room temperature with RVPs (POWV-I and -II) for 1 h. Sera or mAb-virus mixture was added in triplicates to 96-well plates (Costar) containing Vero cell monolayers seeded the day before. After 2 days incubation at 37°C and 5% CO_2_, cells were fixed with 4% paraformaldehyde (Sigma) and washed with PBS. Cell nuclei were stained with Hoechst-33342 (Life Technologies) in a 1:2,000 dilution in PBS. Viral infectivity was measured by automated enumeration of GFP^+^ cells from captured images using a Cytation-5 automated fluorescence microscope (BioTek) and analyzed using the Gen5 data analysis software (BioTek). The half-maximal inhibitory concentration (IC_50_) of plasma and antibodies was calculated using a nonlinear regression analysis with GraphPad Prism software.

### Authentic POWV neutralization assay

Focus-reduction neutralization test (FRNT) experiments were performed as described previously under BSL3 conditions [29]. Briefly, serial dilutions of serum were incubated with 100 FFU of POWV for 1 h at 37°C. Immune complexes were then added to Vero cell monolayers and incubated for 1 h at 37°C prior to the addition of 1% (w/v) methylcellulose in MEM. Following incubation for three days at 37°C, cells were fixed with 1% paraformaldehyde, permeabilized with 0.1% saponin, and stained for infection foci with POWV-16 (1 μg/mL) [49]. Antibody-dose response curves were analyzed using non-linear regression analysis with a variable slope (GraphPad Software).

### Mouse challenge experiments

C57BL/6J mice were purchased from Jackson Laboratories and housed in a pathogen-free animal facility at Washington University in St. Louis. Virus inoculations were performed under anesthesia that was induced and maintained with ketamine hydrochloride and xylazine, and all efforts were made to minimize animal suffering. At 11 weeks of age, mice were administered 100 μL of serum from vaccinated or control mice via intraperitoneal injection. Twenty-four hours later, mice were infected with POWV LB via subcutaneous inoculation in the footpad with 10^2^ FFU of virus. Animals were monitored for mortality and weight loss for 21 days post-infection.

### Measurement of viremia

Serum was collected from mice on day two post-infection. Viral RNA was isolated using the MagMAX viral RNA Isolation Kit (ThermoFisher) and measured by TaqMan one-step quantitative reverse-transcription PCR (RT-qPCR) on an ABI 7500 Fast Instrument. Viral RNA levels are expressed on a log_10_ scale as viral RNA equivalents per mL after comparison with a standard curve produced using serial 10-fold dilutions of viral RNA from known quantities of infectious virus in order to estimate viral burden.

### Structural determination of POWV-EDIII and epitope mapping by NMR spectroscopy

All NMR data were collected at 25°C on Bruker Avance III 600 MHz, 700 MHz or 800 MHz spectrometers equipped with 5 mm cryogenically cooled triple resonance probes. NMR samples consisted of ~500 μM [^15^N]-POWV EDIII in 20 mM phosphate, 5 mM NaCl, pH 6.2 with trimethylsilyl propionate (TSP, shift reference) in 90% H_2_O/10% D_2_O in 3 mm tube or ~1 mM [U-^13^C,^15^N]-POWV EDIII in 20 mM phosphate, 50 mM NaCl, pH 6.2 with TSP in 90% H_2_O/10% D_2_O in 3 mm tube or ~1 mM [U-^13^C,^15^N]-POWV EDIII in 20 mM phosphate, 50mM NaCl, pH 6.2 with TSP in 100% D_2_O in 3 mm tube.

Sequence-specific resonance assignments were obtained using standard triple resonance NMR spectroscopy [71] including ^1^H[^15^N] HSQC, ^1^H[^13^C] HSQC, HNHA, HNCA, HN(CO)CA, HNCO, HN(CA)CO, HNCACB, HN(CO)CACB, (H)CC(CO)NH-tocsy, H(CCCO)NH-tocsy, ^1^H[^15^N] TOCSY-HSQC and HC(C)H-TOCSY. Distance restraints were derived from 3D ^15^N- and ^13^C-edited NOESY-HSQC spectra with a mixing time of 120 ms. All 3D experiments were acquired as non-uniform sampled (NUS) experiments. All NMR data sets were processed on NMRbox [72] with nmrPipe [73] using SMILE reconstruction of the NUS data and analyzed using CCPN Analysis [74]. ^1^H chemical shifts were referenced to TSP and ^15^N and ^13^C chemical shifts were referenced indirectly using proton referencing [75]. 200 structures were calculated using ARIA [76], and energy minimized with XPLOR-NIH 3.2 [77] in explicit water. The 20 lowest energy structures are deposited (PDB 7SGT). The binding epitope of POWV EDIII with the m158.25 Fab was determined by comparing cross peak intensities in the ^1^H-^15^N HSQC and the methyl region of ^1^H-^13^C HSQC between free [U- ^15^N,^13^C] POWV EDIII and complex with m158.25 Fab at a 1:1.1 stoichiometry.

## Supporting information

S1 Fig ELISA reactivity of POWV EDIII and LS-POWV EDIII antisera. (A) IgG subclass profiles of POWV EDIII and LS-POWV EDIII antisera bound to POWV EDIII by ELISA. (B) ELISA reactivity of POWV EDIII and LS-POWV EDIII antisera to monomeric and multivalent POWV EDIII antigen. (C) Cross-reactivity of POWV EDIII and LS-POWV EDIII antisera to related flavivirus EDIIIs. ELISA reactivity of antisera to POWV, TBEV, LGTV, and DENV2 recombinant EDIII was determined. For A-C, groups shown are POWV EDIII monomer (n = 5) and LS-POWV-EDIII nanoparticle (n = 5) at day 56. Full curve ELISAs were performed twice independently in triplicate wells (mean ± SD) and EC_50_ values were determined using non-linear regression analysis. One-way ANOVA with Tukey’s multiple comparison test was done. (*, P < 0.05; **, P < 0.01; ***, P < 0.001, ****, P < 0.0001).
(EPS)

S2 Fig Sequence alignment of POWV strains. Alignment of amino acid sequences of structural polyproteins used for immunogen and reporter virus constructs. EDIII sequence is highlight as indicated. Alignment was generated using ESPript 3.0
(EPS)

S3 Fig POWV-I RVP ELISA. ELISA reactivity of mAbs B2 and B4 against POWV-I RVPs. ELISAs were performed twice independently in triplicate wells (mean ± SD).
(EPS)

S4 Fig Gating strategy for isolation of POWV EDIII-reactive B cells from immunized mice. Representative flow cytometric gating of mouse splenocytes is shown. Cells were filtered for size and granularity. CD3^+^, CD8^+^, F4/80^+^, and Gr-1^+^ cells were excluded and CD19^+^ IgG^+^ POWV EDIII^+^ B cells were collected in individual wells.
(EPS)

S5 Fig Two-phase POWV-80 binning by BLI. Two-phase binding of neutralizing mAbs to POWV EDIII vs. POWV-80 by BLI. Neutralizing mAbs m61.65, m158.25, m158.26 and m158.36 were first bound to a sensor loaded with POWV EDIII and then POWV-80 was added. Representative data from two independent experiments are shown.
(EPS)

S6 Fig ELISA binding curves of neutralizing mAbs against related flavivirus EDIIIs. Full curve ELISAs against POWV, TBEV, LFTV, and DENV2 EDIIIs were performed twice independently in triplicate wells (mean ± SD).
(EPS)

S7 Fig Production and antigenicity of MBP-POWV EDIII mutants. (**A**) SDS-PAGE of purified MBP-POWV EDIII mutants. (**B**) ELISA reactivity of MBP-POWV EDIII mutants to mAb POWV-55. Full binding curves were determined by eight-point serial dilution of POWV-55 against each mutant. Experiment was performed twice independently in triplicate wells.
(EPS)

S8 Fig ELISA binding curves of neutralizing mAbs against MBP-POWV EDIII mutants. Full curve ELISAs against the indicated EDIII mutants were performed twice independently in triplicate wells (mean ± SD).
(EPS)

S9 Fig Two-dimensional 15N-1H heteronuclear single quantum coherence (HSQC) spectra of 15N-labeled POWV EDIII. Residue labels for POWV EDIII assigned peaks are shown.
(EPS)

S10 Fig Monovalent binding kinetics of Fab m158.25 by BLI. Binding of Fab m158.25 to POWV EDIII. A representative data set from two independent experiments is shown.
(EPS)

S1 Table POWV EDIII murine mAb sequence and reactivity profiles. (DOCX)
