# Supplementary material for: A Powassan virus domain III nanoparticle immunogen elicits neutralizing and protective antibodies in mice
Source: PLoS Pathog. 2022 Jun 9;18(6):e1010573. doi: 10.1371/journal.ppat.1010573 (PMC9216602; doi:10.1371/journal.ppat.1010573)
Supplement: S1 Table — (DOCX) [file ppat.1010573.s011.docx]

**S1 Table. POWV EDIII murine mAb sequence and reactivity profiles**

| **mAb** | **V-gene** | **CDR lengths** | **CDR3** | **POWV EDIII EC_50_ (nM)** |
| --- | --- | --- | --- | --- |
| m61.4 | IGHV1-54 | 8.8.10 | CARDGSYAMDYW | 7.6 |
|  | IGKV8-28 | 12.3.9 | CLSDHSYPFTF |  |
|  |  |  |  |  |
| m61.23 | IGHV2-9 | 8.7.11 | CARLYYYGVFDYW | 5.5 |
|  | IGKV8-24 | 12.3.9 | CQQHYSTPLTF |  |
|  |  |  |  |  |
| m61.37 | IGHV1-18 | 8.8.9 | CARWRYGPDYW | 2.9 |
|  | IGKV8-19 | 12.3.9 | CQNDYSYPLTF |  |
|  |  |  |  |  |
| m61.39 | IGHV1-69 | 8.8.12 | CTRSLGLRSFFDYW | 5.7 |
|  | IGKV2-109 | 11.3.9 | CAQNLELPWTF |  |
|  |  |  |  |  |
| m61.53 | IGHV2-6-5 | 8.7.15 | CAKHGGITSVRYAMDYW | 1.7 |
|  | IGKV3-7 | 10.3.9 | CQHSWEIPYTF |  |
|  |  |  |  |  |
| m61.65 | IGHV1S29 | 8.8.9 | CARSDYGFAYW | 2.0 |
|  | IGKV4-55 | 5.3.9 | CQQWSSYPYTF |  |
|  |  |  |  |  |
| m61.80 | IGHV3-2 | 9.7.13 | CAREDYGYDVYFDYW | 12 |
|  | IGKV10-96 | 6.3.9 | CQQGKTLPLTF |  |
|  |  |  |  |  |
| m61.89 | IGHV1-54 | 8.8.10 | CARSGSVEFDYW | 2.4 |
|  | IGKV6-15 | 6.3.9 | CQQYNSYPFTF |  |
|  |  |  |  |  |
| m158.18 | IGHV1-7 | 8.8.8 | CAHGPWFAYW | 6.0 |
|  | IGKV8-19 | 12.3.9 | CQNDYSYPLTF |  |
|  |  |  |  |  |
| m158.25 | IGHV1-7 | 8.8.14 | CARGGYDFDGYYFDYW | 8.8 |
|  | IGKV10-96 | 6.3.9 | CQQGKTLPPTF |  |
|  |  |  |  |  |
| m158.26 | IGHV1-54 | 8.8.10 | CARDDSTLLGYW | 2.9 |
|  | IGKV6-15 | 6.3.9 | CQQYNSYPFTF |  |
|  |  |  |  |  |
| m158.36 | IGHV1-67 | 8.8.9 | CVRGPYAMDYW | 3.6 |
|  | IGKV3-5 | 10.3.9 | CQQDNEVPWTF |  |
|  |  |  |  |  |
